# Effect of fluoride mouthrinses and stannous ions on the erosion protective properties of the *in situ* pellicle

**DOI:** 10.1038/s41598-019-41736-7

**Published:** 2019-03-29

**Authors:** A. Kensche, E. Buschbeck, B. König, M. Koch, J. Kirsch, C. Hannig, M. Hannig

**Affiliations:** 1 Clinic of Operative and Pediatric Dentistry, Medical Faculty Carl Gustav Carus, TU Dresden, Fetscherstr. 74, D-01307 Dresden, Germany; 2 Clinic of Operative Dentistry, Periodontology and Preventive Dentistry, University Hospital, Saarland University, Building 73, D-66421 Homburg/Saar, Germany; 3 Physical Analytics, INM – Leibnitz Institute for New Materials, D-66123 Saarbrücken, Germany

## Abstract

The particular feature of this study is the investigation of effects of pure fluoride- or stannous ions based mouthrinses on the erosion protective properties and the ultrastructure of the *in situ* pellicle (12 volunteers). Experimental solutions were prepared either from 500 ppm NaF, SMFP, AmF or SnF_2_ or 1563 ppm SnCl_2_, respectively. After 1 min of *in situ* pellicle formation on bovine enamel slabs, rinses with one of the preparations were performed for 1 min and intraoral specimens’ exposure was continued for 28 min. Native enamel slabs and rinses with bidestilled water served as controls. After oral exposure, slabs were incubated in HCl (pH 2; 2.3; 3) for 120 s and kinetics of calcium- and phosphate release were measured photometrically; representative samples were analysed by TEM and EDX. All mouthrinses reduced mineral loss compared to the native 30-min pellicle. The effect was pH-dependent and significant at all pH values only for the tin-containing mouthrinses. No significant differences were observed between the SnF_2_- and the SnCl_2_-containing solutions. TEM/EDX confirmed ultrastructural pellicle modifications. SnF_2_ appears to be the most effective type of fluoride to prevent erosive enamel demineralisation. The observed effects primarily have to be attributed to the stannous ions’ content.

## Introduction

In the oral cavity, all chemical, physical and microbial processes at the tooth surface involve molecular interactions with the acquired dental pellicle^1^. Transmission electron microscopic analyses have thoroughly described the characteristic ultrastructure of this two-layer proteinaceous *in situ* pellicle^1,2^. It consists of a 10–20 nm thick electron dense basal layer and a more globular or granular outer layer which is generally more susceptible to alterations by ambient noxae^1^. Evidently, even after short formation times, the *in situ* pellicle has a certain protective effect against erosive demineralisation of the dental hard tissues^3–5^. An optimisation of these erosion protective pellicle properties by oral prophylactic measures *in situ* often correlates with an enhanced electron density of the pellicle^6–8^. The attack of acids at the tooth surface as well as the retention of calcium and phosphate in the pellicle layer might be altered due to pellicle modifications^3^.

Recent *in vitro* and *in situ* investigations have shown, that the application of different fluoride containing preparations might have an influence on the pellicle layer or the pellicle’s ultrastructure, respectively^7,9^. It has been suggested, that the fluoride bound cations interfere with the adsorption processes of salivary proteins at the tooth surface^7,9^. Ideally, this would promote the formation of a more acid-resistant pellicle layer. *In situ* investigations have yielded corresponding results after simple mouthrinses with a sodium fluoride (NaF)-, amine fluoride (AmF)- and stannous chloride (SnCl_2_) containing customary preparation^7^. In comparison, a purely NaF-based mouthrinse appeared to facilitate protein re-adsorption to the demineralised enamel surface after acid exposure. This might rather contribute to a remineralising effect^7,10,11^. Especially stannous ions significantly improved the erosion preventive effect of fluoride containing prophylactic measures *in vitro* and *in situ*^7,12–16^. One explanation could be the strengthening of the pellicle’s acid resilience^7^. Algarni *et al*. suggested an additive effect of stannous- and fluoride ions in cross-linking pellicle proteins *in vitro*^9^. The necessity of a combination of stannous ions with fluoride components to evolve their erosion preventive effect has not yet been analysed *in situ*.

*In situ* investigations with pure active agents at the same concentration are necessary to further understand the potential effects of fluorides and different cations at the tooth surface. So far, certain erosion-preventive effects of common fluoride preparations such as sodium- or amine fluoride have primarily been attributed to the formation of protective CaF_2_-like precipitates at the tooth surface^17^. The confirmative detection of these material deposits *in situ* is limited and strong erosive noxae appear to have a virtually unhampered impact on the dental hard tissue^7,10,18,19^. In comparison, stannous ions are believed to induce the deposition of a more resilient and constant tin- and fluoride enriched layer on the enamel surface^12,20,21^. Several studies have aimed to measure the level of stannous ions on dental hard tissue after different treatment protocols^12,22,23^. However, a clear description of a metal-enriched coating has only been performed by energy-dispersive X-ray spectroscopy under *in vitro* conditions^12^.

It must be assumed that pellicle modifications notably contribute to the variable erosion preventive potential of different fluoride containing mouthrinses. In this study, we hypothesized that the type of fluoride has a pronounced impact on the ultrastructure and the erosion preventive properties of the *in situ* pellicle. In order to test these hypotheses, experimental mouthrinses were prepared from pure fluoride components in one concentration (500 ppm). *In situ* pellicle samples were collected with or without being rinsed and they were subjected to transmission electron microscopic- and colorimetric analyses before and after short term acid attack.

## Methods

### Test solutions

All tested experimental rinsing solutions were individually prepared from the pure (fluoride) substances on the day of their application (Table 1). An uniform concentration of 500 ppm fluoride was chosen as it is found in different customary mouthrinses^7^. The investigated fluoride compounds included: pure sodium fluoride (NaF), sodium monofluorophosphate (SMFP), 33% amine fluoride (AmF) in propylene glycol and stannous fluoride (SnF_2_). For every testing 100 ml rinsing solution were prepared, dissolving the substances in distilled water by vortexing at room temperature. Additionally, the concentration of tin included in 100 ml of a 500 ppm SnF_2_ containing solution was calculated on the basis of the molar masses of fluoride and tin. Stannous ions have a molar mass of 118 g/mole while fluoride has a molar mass of 19 g/mole which yields 118/38 = 3.1 for SnF_2_. According to the defined concentration of 500 ppm fluoride for all investigated fluoride-based preparations a concentration of 1563 ppm stannous ions was calculated and an experimental SnCl_2_ containing mouthrinse was prepared. At baseline, the pH-values of all mouthrinses were measured with a pH meter (Mettler-Toledo GmbH, Gießen, Germany).

**Table 1:** Summary of specific (fluoride) substances and the resulting manually produced mouthrinses.

Pure Substance	Concentration	Solution volume	Weighed quantity	Measured pH value
Sodium fluoride (Ferdinand Kreutzer Sabamühle GmbH, Nürnberg, Germany)	500 ppm fluoride concentration	100 ml	110,6 mg	7.1
Sodium monofluorophosphate (Omya Schweiz AG, Oftringen, Switzerland)			379,0 mg	6.6
Permafluor = amine fluoride 33% (Permcos GmbH, Arisdorf, Switzerland)			1967,69 mg	3.6
Stannous fluoride (Honeywell Specialty Chemicals GmbH, Seelze, Germany)			206,3 mg	4.5
Stannous chloride dehydrate (Honeywell Specialty Chemicals GmbH, Seelze, Germany)	1563 ppm tin concentration		297,1 mg	3.5

### Experimental setup for *in situ* investigations

**Figure 1:**
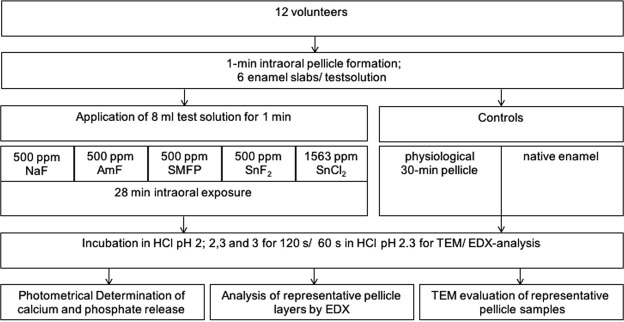
Flowchart of the experimental setup.

The present investigation involved both *in situ* as well as subsequent *ex situ*/*in vitro* methods^6,7,24^ (Fig. 1). Any intraoral material exposure had been approved by the ethics committee of the Medical Faculty, Technische Universität Dresden, Germany (vote: EK 475112016) and all experiments were carried out in accordance with the relevant guidelines. Twelve healthy non-smoking volunteers (6 M/6 F) aged from 22 to 29 had given their informed written consent about participation in the study. Initial dental examination confirmed that none of them showed signs of caries, periodontal disease, severe non-carious dental hard tissue defects or an unphysiological salivary flow rate.

The investigated *in situ* pellicle samples were obtained on intraorally exposed bovine enamel slabs from 2-year-old cattle (5 mm diameter, 2 mm thickness, 19,63 mm^2^ surface area)^6–8^. In terms of chemical composition and microhardness bovine enamel resembles its human counterpart and is therefore a suitable substitute^25,26^. Recent investigations have clarified, that even though the protein composition of 120 min pellicles formed on human or bovine enamel vary, no significant differences were detected regarding the presence of characteristic pellicle proteins such as Histatin-1, Statherin or Lysozyme^27^. Bovine enamel slabs have been used in several previous studies based on the same methods and the same *in situ* model. A number of publications serve as references. The specimens were prepared as follows: all slabs’ surfaces except the outer enamel surface were sealed with the total-etch based Optibond FL adhesive system according to the manufacturers guidelines (Etching gel, DMG, Hamburg, Germany; OptiBond FL, Kerr, Karlsruhe, Germany)^7,28^. The remaining enamel surface was wet-grinded and polished with up to 4000 grid abrasive paper and the resulting smear layer was removed by steam jet and ultrasonication with 3% NaOCl for 3 min. If a final microscopic examination showed no signs of structural enamel alterations, the slabs were washed twice in destilled water, disinfected in 70% ethanol, both activated by ultrasonication and were stored in distilled water for a maximum of 24 h before intraoral exposure.

Eventually, six enamel slabs at a time were fixed in small buccal cavities of individually adjusted upper jaw splints by polyvinylsiloxane impression material (Provil novo light regular set, Heraeus Kulzer, Germany), so that only the enamel surface was exposed to the oral environment (region 14–16 and 24–26). All *in situ* experiments were scheduled between 7 and 12 am in the morning. The different mouthrinses were tested on separate days to guarantee a wash-out period of at least 48 hours. In between, the participants performed their regular oral hygiene. Only two hours before any test period, they were instructed to brush their teeth without toothpaste and to refrain from any intake of food or drinks other than water afterwards.

In accordance with previous studies, the specimens’ exposure *in situ* covered a total period of 30 min^6,7,28^. After 1 min of pellicle formation, rinses with 8 ml of one of the freshly prepared mouthrinses were performed for 1 min and the splints remained intraorally for another 28 min. Unrinsed specimens with a 30-min *in situ* pellicle as well as native enamel slabs served as controls. Afterwards the specimens were rinsed thoroughly with distilled water and were quickly removed from the splints to be subjected to the erosive challenge *in vitro*.

### Determination of *in vitro* erosion

Different erosive challenge was simulated for 120 s *in vitro* with hydrochloric acid of pH 2, 2.3 and 3 and the corresponding calcium- and phosphate releases were quantified by a well-established photometric double assay^6,29^. Therefore, the pellicle covered enamel slabs were embedded at the bottom of a 2 ml Eppendorff cup (Provil novo light regular set, Heraeus Kulzer, Germany). For every experimental condition 2 enamel slabs were investigated. One milliliter of the acid solution was applied and constantly circulated by pumping with a 100 µl pipette (1 lift/s) to provide an even pH-value. Every 15 s, 100 µl of the acid were removed for photometric analysis and replaced immediately by 100 µl of fresh acid. The dissociated calcium could be determined on the basis of the Arsenazo III method (Fluitest ®, Ca-A-II, analyticon, Lichtenfels, Germany) while released phosphate interacted characteristically with malachite green^24^. The intensity of both resulting colored complexes was quantified at λ = 650 nm according to standard curves. All measurements were performed as triplicate tests, with the average adsorption being calculated.

### Statistics

All data were statistically processed by SPSS 21.0 (IBM, Ehningen, Germany). The calcium- and phosphate release measurements after 120 s of incubation in HCl were averaged from 2 enamel slabs per experimental condition and subject. A normal distribution of the data was disproved by the Shapiro-Wilk test. Accordingly, the Kruskal-Wallis test was performed to detect significant influences of the mouthrinses on calcium- and phosphate loss during HCl exposure. Additional pairwise comparison was achieved by adoption of the Mann-Whitney U test. The Bonferroni-Holm-adjusted level of significance was set to p ≤ 0.001.

### Electron microscopic examination

Besides quantifying an acid induced ion-dissociation from the enamel surface, the present study additionally aimed to investigate the pellicle’s ultrastructure under the influence of the different test solutions. Therefore, *in situ* experiments were carried out as described above and 2 exemplary 30-min pellicle samples of every treatment were subjected to transmission electron microscopic investigation. After the slabs had been removed from the splints they were carefully rinsed with distilled water and either fixed in 1.5% formaldehyde −2.5% glutaraldehyde solution for 1 h at 4 °C or, prior to this, were incubated in HCl (pH 2.3) for 60 s. The fixed samples were thoroughly washed in 0.1 M cacodylate buffer and 1 ml of 1% osmium tetroxide was applied for 2 h to enhance the visualization of organic structures. An ascending ethanol-series was completed before embedding the dehydrated pellicle samples in Araldite CY212 (Agar Scientific Ltd, Stansted, United Kingdom). After decalcification of the remaining enamel by 0.1 M HCl, the samples were reembedded in Araldite. Ultrathin sections of the pellicle samples, cut in series with an ultramicrotome (Ultracut E, Reichert, Bensheim, Germany), were placed on pioloform coated copper grids (Plano, Wetzlar, Germany) and contrasted with uranyl acetate and lead citrate. A TEM TECNAI 12 Biotwin (FEI, Eindhoven, The Netherlands) was used for the transmission electron microscopic analysis at magnification of up to 49,000-fold.

In order to investigate the traceability of stannous ions in the pellicle samples, transmission electron microscopy coupled energy dispersive X-ray spectroscopy was additionally performed on the ultrathin pellicle sections. Several sections of the specimens and particularly electron dense areas were analyzed by a JEOL JEM-2100 (LaB6) (JEOL, Nieuw-Vennep, The Netherlands) which was fitted with an EDS detector. Based on the results from recent investigations only samples that had been exposed to the tin containing mouthrinses were included^7^.

## Results

### Acid derived ion dissolution

**Figure 2:**
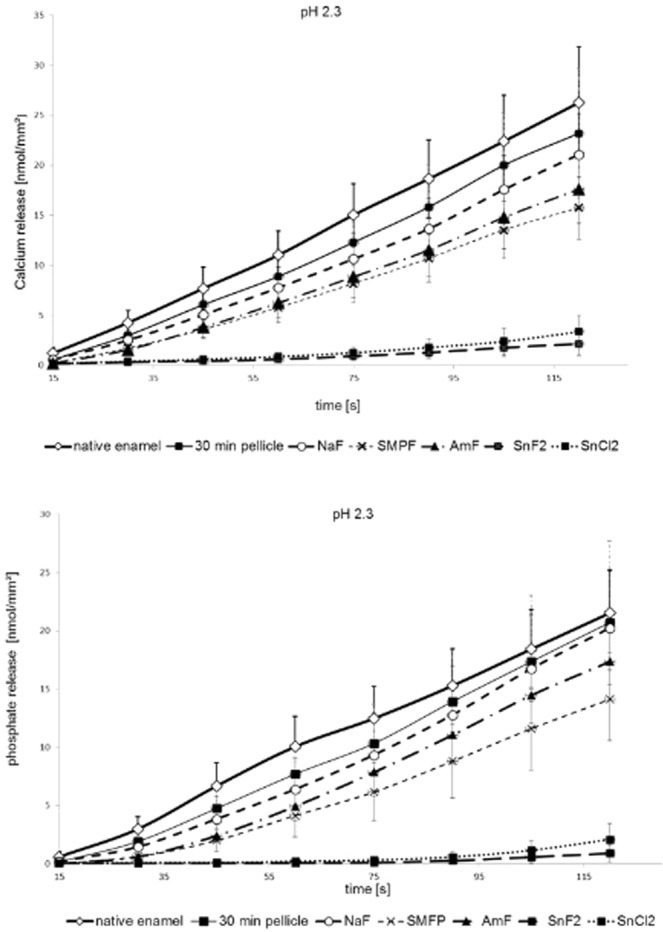
Kinetics of calcium and phosphate release during 120-s incubation of pellicle covered enamel slabs in HCl, representatively shown for pH 2.3. Data include 30-min *in situ* pellicles modified by one of the investigated fluoride- and stannous ions containing mouthrinses for 1 min as well the physiological 30-min *in situ* pellicle and native enamel specimens which served as controls. In all experimental groups ion dissolution occurred linearly over the incubation time. A significant enhancement of the pellicle’s demineralisation preventing effect could only be achieved by the stannous ions containing preparations. n = 12 subjects, n = 24 enamel samples per subgroup, mean values ± standard deviation.

**Figure 3:**
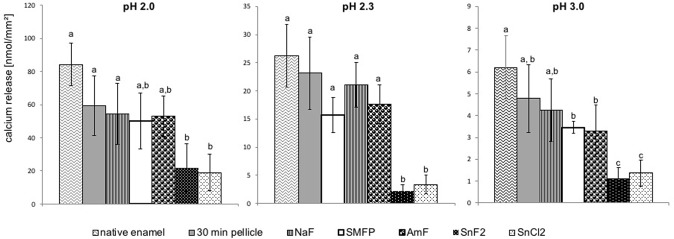
Cumulative calcium release from enamel slabs after 30 min of pellicle formation with and without having been exposed to one of the investigated fluoride- and stannous ions containing mouthrinses *in situ* and incubation in HCl (pH 3.0, 2.3, 2.0) for 120 s. Specimens without pellicle served as controls; n = 24 samples per subgroup. Data significantly different from each other are marked with different letters. *In situ* pellicle formation reduced calcium release at all pH-values. This effect was slightly enhanced by fluoride application. A significant increase (p < 0.001 after Bonferroni-correction) of erosion preventive pellicle properties at all pH values was only observed after application of the stannous ions containing mouthrinses (Kruskal-Wallis-/Mann-Whitney-U-test with Bonferroni-correction).

**Figure 4:**
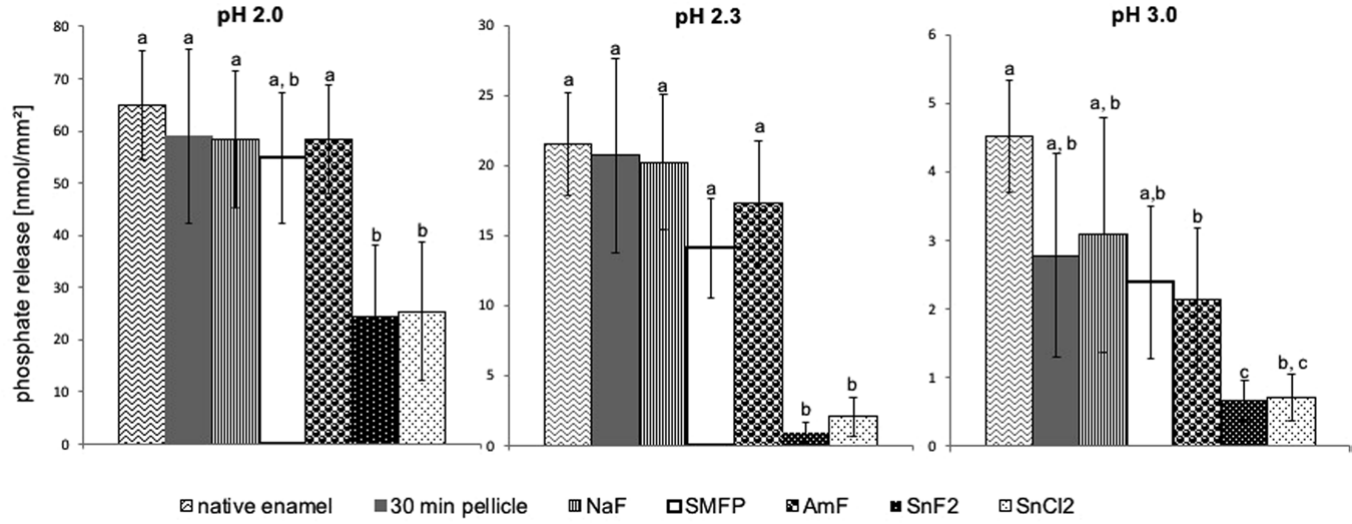
Cumulative phosphate release of enamel slabs after 30 min of pellicle formation with and without having been exposed to one of the investigated fluoride- and stannous ions containing mouthrinses *in situ* and incubation in HCl (pH 3.0, 2.3, 2.0) for 120 s. Specimens without pellicle served as controls; n = 24 samples per subgroup. Data significantly different from each other are marked with different letters. Most investigated mouthrinses had no significant influence on the native 30-min pellicle. However, a significant decrease of phosphate dissolution was determined after application of the stannous ions containing mouthrinses (Kruskal-Wallis-/Mann-Whitney-U-test, p < 0.001 after Bonferroni-correction).

A total number of 432 intraorally exposed enamel slabs were subjected to the photometric determination of acid induced calcium- and phosphate release *in vitro*. The brief incubation of all enamel specimens in HCl (pH 2, 2.3. or 3) lead to a detectable release of calcium and phosphate into the solution. Characteristically, the extent of this acid induced demineralisation process depended on the pH value and increased quite linearly over the exposure time as depicted exemplarily for pH 2.3 in Fig. 2. Furthermore, statistical evaluation was performed for the cumulative mineral loss over 120 s (Figs 3 and 4). In comparison to native enamel, even only the formation of the proteinaceous 30-min *in situ* pellicle reduced the cumulative amount of released calcium after 120 s by 23% at pH 3.0, 12% at pH 2.3 and 29% at pH 2.0. Similarities were confirmed for the phosphate measurements (Figs 3 and 4). All investigated mouthrinses additionally decreased the detected amount of calcium and phosphate in solution, but pairwise comparison revealed marked differences between the tested substances to significantly improve the pellicle’s erosion protective properties. Although the relative percentage of ions’ dissolutions’ inhibition clearly depended on the particular mouthrinse, it appeared to be rather consistent in between all pH values. Comparing all tested fluoride substances, the mouthrinse prepared of 500 ppm sodium fluoride seemed to have the least pronounced impact on the physiological pellicle. Furthermore, neither the application of sodium monofluorophosphate nor rinsing with a 500 ppm pure amine fluoride solution significantly improved the protective pellicle properties at any of the investigated pH values (Figs 3 and 4). Very much on the contrary, the 500 ppm stannous fluoride based solution inhibited the acid induced calcium release by another 59% at pH 3.0, 80% at pH 2.3 and 47% at pH 2. This did not only indicate a significant beneficial impact on the physiological pellicle but also meant a significant superiority to inhibit erosion derived ion release, when compared to the other fluoride containing mouthrinses (p < 0.001) (Fig. 3).

In all experimental groups, incubation of the enamel slabs in HCl also caused the detectable dissolution of phosphate (Figs 2 and 4). Due to the chemical composition of enamel, generally less phosphate than calcium was released^30^. Under acidic conditions phosphate anions are dominant in form of H_3_PO_4_ and H_2_PO4^−^ ^30^. Compared to the efficacy of the native pellicle or the other fluoride containing mouthrinses to reduce erosive phosphate dissolution, only the SnF_2_ based mouthrinse could significantly decrease the traceability of dissociated phosphate (Fig. 4).

In order to differentiate the effects of fluoride or stannous ions on the inhibition of erosive ions’ release, respectively, a mouthrinse containing an equal amount of stannous ions without fluoride was included in this study. Obviously, no relevant difference was detected between the two tin-containing preparations, neither regarding calcium- (SnF_2_ vs SnCl_2_ = pH 3: <4%, pH 2.3: <5%, pH 2: >3%), nor regarding phosphate release (SnF_2_ vs SnCl_2_ = pH 3: <1%, pH 2.3: <6%, pH 2: <1%).

### The pellicle’s ultrastructure

**Figure 5:**
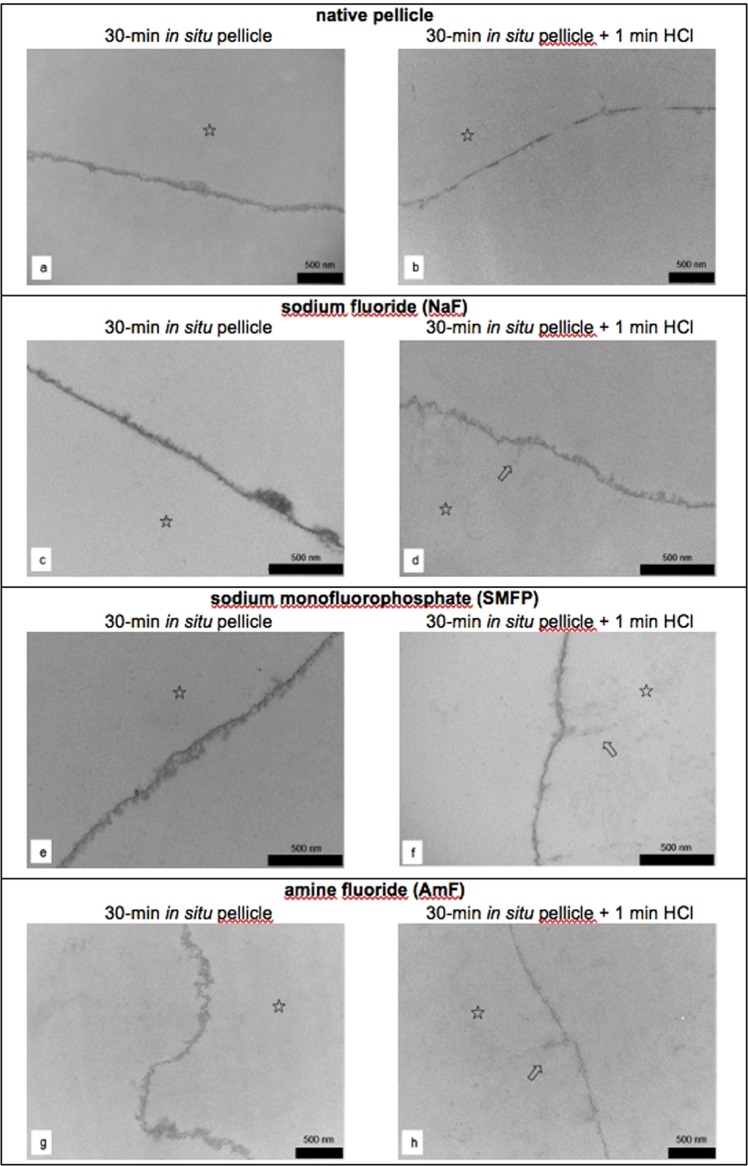
Representative TEM – images of 30-min *in situ* pellicle samples without (**a**,**b**) and with pretreatment by different common fluorides containing experimental mouthrinses (**c**–**h**) before (**a**,**c**,**e**,**g**) and after incubation in HCl (pH 2.3) for 1 min (**b**,**d**,**f**,**h**). The characteristic ultrastructure of the thin continuous proteinaceous pellicle was detected after 30 min of enamel specimens’ exposure *in situ*. None of the mouthrinses appeared to have an influence on the density or thickness of the formed pellicle. In all cases, HCl-incubation caused the partial protein detachment or dissolution of the pellicle, respectively (**b**,**d**,**f**,**h**). However, after previous fluoride application eroded surface defects appeared to immediately be recovered by protein adsorption (see ⇧ in **d**,**f**,**h**). Please note that the former enamel site is marked with an asterisk as it was removed during the preparation process. Original magnification: 30,000–49,000 fold.

**Figure 6:**
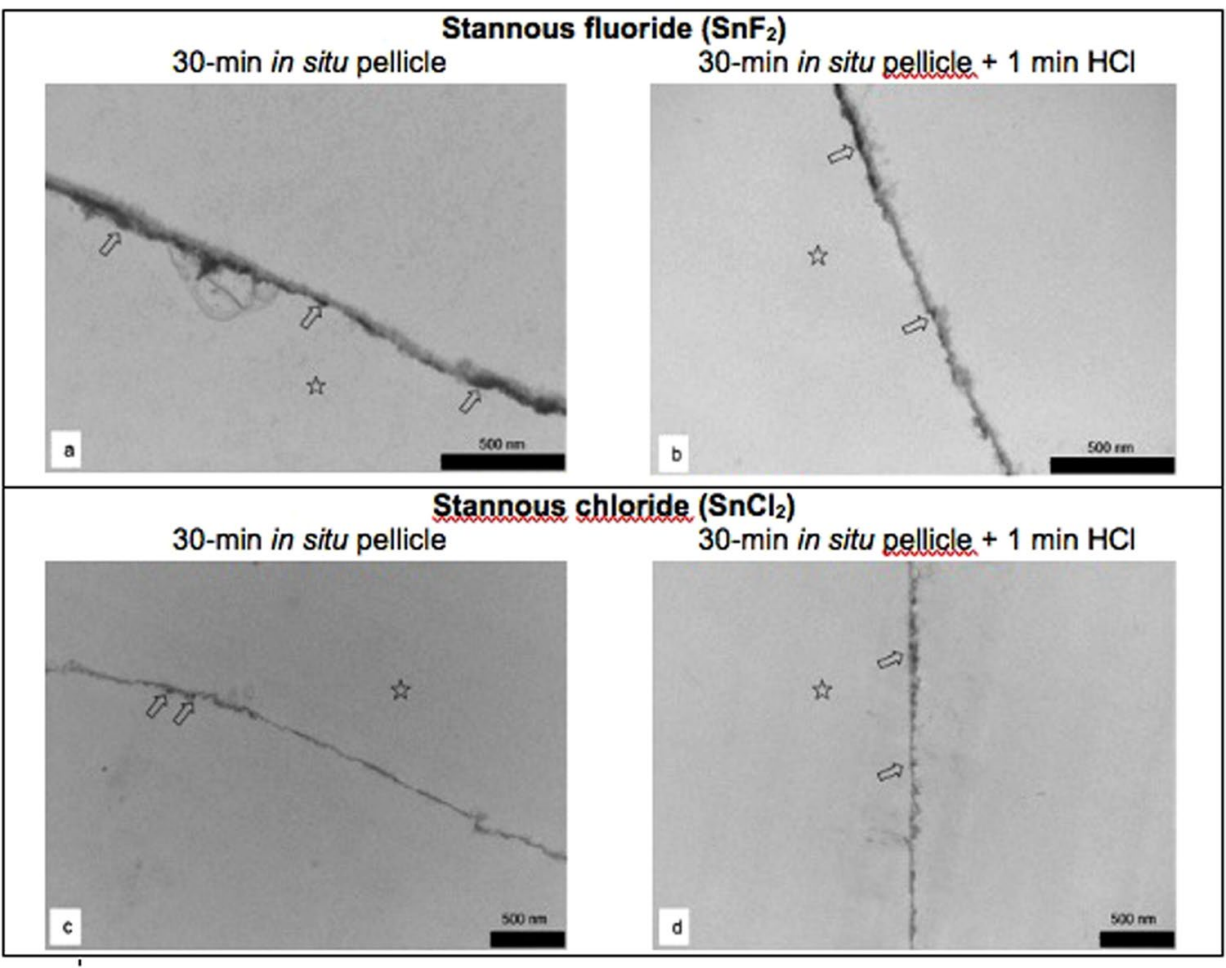
Representative TEM – images of 30-min *in situ* pellicle samples after application of stannous fluoride- (**a**,**b**) or stannous chloride (**c**,**d**) based mouthrinses before and after incubation in HCl (pH 2.3) for 1 min. In contrast to all other fluoride containing mouthrinses (see Fig. 5), availability of stannous ions appeared to enhance the pellicle’s resistance against acidic dissolution. The basic layer of the 30-min *in situ* pellicle revealed in part a higher electron density with a verifiable accumulation of tin-compounds that also remained after incubation in HCl (⇧). No signs of enamel demineralisation or pellicle dissolution were observed after acid exposure (**b**,**d**). Please note that the former enamel site is marked with an asterisk as it was removed during the preparation process. Original magnification: 30,000–49,000 fold.

In order to investigate ultrastructural characteristics of initial pellicle formation under the influence of the different (fluoridated) mouthrinses *in situ*, TEM - analyses were performed on exemplary 30-min *in situ* pellicle samples before and after incubation in HCl pH 2.3 for 1 min (Figs 5 and 6). As expected, the physiological 30-min *in situ* pellicle samples showed the characteristic thin but electron dense and continuous protein accumulation at the former enamel surface (5a). However, pellicle dissolution and desintegration derived from acid exposure (Fig. 5b). After the application of the 500 ppm NaF-_,_ SMFP- or AmF containing preparations, the appearance of the 30-min *in situ* pellicle remained rather consistent. In contrast, pellicle formation under the availability of stannous ions or stannous fluoride, respectively, slightly increased the electron density of the basal pellicle layer (Fig. 6). However, more distinct differences between the mouthrinses could be seen after the specimens’ acid exposure. Both, the pellicle’s ultrastructure as well as, indirectly, the former enamel surface showed indications of acid induced lesions, if the applied mouthrinses were based on NaF, SMFP or AmF. Incubation in HCl (pH 2.3) for 1 min caused the partial protein desorption or dissolution of the pellicle samples, respectively. As observed earlier, the superficially eroded enamel surface was recovered by adsorbed proteins (Fig. 5)^7^. This infiltration of the demineralisation defects was induced by all common fluoride containing preparations (Fig. 5). Most remarkable results were gained from the TEM-analysis of acid exposed 30-min *in situ* pellicle samples that were modified by the two stannous ions containing mouthrinses (Fig. 6). Even after incubation in HCl (pH 2.3) for 1 min, rather unaffected continuous and electron dense pellicle layers and no signs of enamel infiltration were detected (Fig. 6). The particularly electron dense sections still appeared to be intact which was possibly due to the remaining accumulation of tin compounds in the pellicle layer.

Aiming to clarify this assumption, additional energy-dispersive X-Ray spectroscopy was performed. The traceability of Sn was confirmed in all of the investigated 30-min *in situ* pellicle samples, no matter which Sn-containing mouhrinse was applied. Even though the method does not allow specific quantitative evaluation, no notable differences were observed regarding the distribution or signal intensity of traceable stannous compounds in the pellicle. Furthermore, after acid exposure, tin was equally detectable in the corresponding pellicle samples. In all cases, the measurable signal for Sn presence did not change considerably. EDX-analysis was additionally performed on several of the pellicle samples that were formed under the influence of the Sn-free fluoride containing mouthrinses and no Sn was detected. These results were in line with our previous observations^7^.

## Discussion

For the first time, a study is presented here that investigated the effect of pure quality fluoride components on the pellicle ultrastructure and the erosion relevant pellicle properties *in situ*. Additionally, a fluoride free stannous ion containing mouthrinse was included. Preventing demineralisation before promoting remineralisation is an essential preliminary step to retain the dental hard tissue structure under erosive conditions^10,31^. The broad diversity of study designs and investigated preparations published in the field of fluorides in erosion makes it difficult to gain a clear understanding of the active substances’ mode of action at the tooth surface^14,32–34^. To counter this, the experimental fluoride containing mouthrinses were prepared at one concentration of 500 ppm. This specific concentration was chosen because it is found in customary mouthrinses. Furthermore, we have recently shown that a customary 500 ppm NaF/AmF/SnCl_2_ preparation had a notable impact on *in situ* pellicle formation^7^. A fluoride free tin containing solution was prepared based on the molar masses of the respective ions in a 500 ppm SnF_2_ – solution (1563 ppm SnCl_2_).

Fluoridated mouthrinses are a common supplement of daily oral hygiene measures. Since pellicle formation occurs immediately on all dental hard tissues in the oral cavity, the clinical situation of brushing the teeth and additionally applying a mouthrinse was simulated in the present study by allowing one minute of pellicle formation. The intraoral exposure time of the enamel slabs was limited to 30 min because there is some evidence from the literature that the pellicle’s potential to prevent erosive enamel demineralization does not significantly change after 30 min, 1, 6, 12 or 24 h of pellicle formation^3,35^.

All fluoride components typically contained in dental prophylactic agents were included in the study. The uniform handling of the substances allowed their reasonable comparability under *in situ* conditions. Due to the purity of the mouthrinses, a clear correlation could be drawn between the individual active substance and the observed effects. For this purpose, adaption of the pH values has deliberately been omitted. In this case, any influence of further additives could be excluded. The acidic pH values of most of the experimental preparations were comparable to common customary mouthrinses^7^. Certainly, the different pH levels of the applied solutions will have contributed to their effects at the tooth surface. However, regarding the pH value und the results of the AmF- (3.6) in comparison to the SnF_2_- (4.5) and SnCl_2_ (3.5) based mouthrinse, no unequivocal conclusion could be drawn between the erosion preventive potential and the effect of the acidity of the applied mouthrinse. The main focus of the present study was to investigate pure fluoride- and stannous ions containing preparations without the addition of any neutralising or acidic components *in situ*.

Naturally, the dynamics inside the oral cavity in terms of salivary clearance and biofilm remodelling processes at the tooth surface cannot fully be simulated *in vitro*^9,31^. Therefore, the combination of a functional and structural *in situ* pellicle investigation yields important information on underlying scientific mechanisms as well as the clinical relevance of the applied mouthrinses in erosion prevention. The colorimetric determination of erosive mineral loss is a well-recognized method to detect initial demineralisation processes at the tooth surface, such as provoked by acidic drinks^29^. According to Shellis *et al*. dental enamel reacts to acidic attack as follows: [Ca_8.9_Na_0.3_Mg_0.14_K_0.01_] [(PO_4_)_5.1_(HPO_4_)_0.4_(CO_3_)_0.5_] [(OH)_1.08_(CO_3_)_0.05_Cl_0.1_] + (1.78 + 3x + 2y + z)H^+^ → 8.9 Ca^2+^ + 0.3 Na^+^ + 0.14 Mg^2+^ + 0.01 K^+^ + 0.1 Cl^−^ + xH_3_PO_4_^3−^ + yH_2_PO_4_^−^ + zHPO_4_^2−^ + 1.1 CO_2_(gas) + 2.18 H_2_O. In case of the dissociated phosphate, lowering of pH-value (pH 2.3, 2) will have caused a shift from H_2_PO4^−^ to H_3_PO_4_. The results of the acid induced calcium- and phosphate release measurements as well as the ultrastructural analysis of representative pellicle samples give sufficient reason to suppose that singular NaF-, AmF, or SMFP- application do not significantly affect the erosion preventive pellicle properties, at least under the experimental conditions of the present study. Dissociated calcium- and phosphate levels increased linearly with the drop of pH value and were only slightly lower than after unmodified pellicle formation.

The ultrastructure of neither of the 30-min *in situ* pellicle samples after NaF-, AmF- or SMFP rinsing showed any noticeable differences regarding their thickness or electron density. It must be concluded, that the formation of a demineralisation barrier due to single fluoride application, either in form of CaF_2_-precipitates at the tooth surface or pellicle condensation, would be very limited. However, similar to previous TEM observations, protein attachment at the eroded enamel surface seemed to be enhanced by the fluoride based mouthrinses if compared to the control^7,10^ (Fig. 5). Most likely, this protein adsorption contributes to the remineralising action of NaF, AmF or SMFP^36^, respectively. Even though SMFP does not contain any free fluoride, it also appeared to support protein attachment at the eroded enamel surface. Thereby, the timing of specific fluoride application before or after erosive attack should be reconsidered in clinical practice.

In comparison to all other mouthrinses, both stannous salts based preparations (SnF_2_ and SnCl_2_) have significantly reduced the acid induced demineralisation of the enamel surface. This further reinforces the erosion preventive effect of stannous ions containing prophylactic agents^7,32,37^. Unlike many other studies, the treatment protocol was deliberately designed to base on one-off applications of the mouthrinses. Consequently, initial modifications of bioadhesion processes could be detected more reliably. According to the general understanding, stannous ions induce the formation of a stable coating at the tooth surface consisting of Sn_2_(PO_4_)OH, Sn_3_F_3_PO_4_ and Ca(SnF_3_)^21^. Interestingly, the fluoride content appeared to be negligible for the demineralisation inhibiting effect in the present study. Neither the amount of measurable calcium and phosphate nor the ultrastructural appearance of the pellicle after HCl incubation (pH 2.3) differed considerably between the two Sn^2+^ containing mouthrinses. Few previous publications on the topic have mentioned potential molecular interactions of stannous ions with pellicle components^7,9,33,38,39^. In contrast to monovalent Na+ ions, divalent Sn^2+^ cations might crosslink pellicle proteins and increase the adsorption of specific proteins such as mucins and albumin at the enamel surface^9,38,39^.

Clearly, the TEM images further confirm the rather resistant adsorption of tin compounds into the pellicle layer which will contribute to the erosion preventive efficacy of the metal cation *in situ*. Even though representative 30-min pellicle samples formed after the usage of the SnF_2_- or SnCl_2_ containing mouthrinses did not differ distinctively from the native control, parts of their basic pellicle layer still appeared to be more electron dense. Obviously, both SnF_2_- and SnCl_2_-modifications increased the pellicle’s resistance against acidic dissolution and no signs of eroded enamel were detected (Fig. 6). Further investigation of several of those electron dense areas by TEM-coupled EDX-analysis confirmed an accumulation of stannous compounds. Considering the distribution of these electron dense sections throughout the analysed pellicle samples, no distinct difference could be observed between the two types of stannous ions providing mouthrinses. Similarities were seen after acid exposure, comparable proportions of Sn were detected in all investigated samples. Eventually, it must be concluded that the significant erosion preventive potential of stannous ions containing mouthrinses is predominantly due to the verifiable adsorption of stannous ions into the pellicle layer.

The presented data show, that stannous ions provide a reasonable potential to effectively be used in erosion preventing prophylactic strategies. Particularly the initial enamel demineralisation induced by severe erosive attack (pH < 3) can be reduced significantly, if pellicle formation occurs under the availability of Sn^2+^-cations. To the best knowledge of the authors no other *in situ* study has so far unequivocally elucidated the significant fluoride independent erosion preventive effect of Sn^2+^ in form of pure stannous chloride. Ultrastructural and compository pellicle modifications must be considered as important influences of stannous ions containing prophylactic agents *in situ*. The eligibility of fluorides to be regarded as the goldstandard in daily oral hygiene is still unopposed as they have multiple influences in caries- and biofilm control. However, individual prophylactic measures have to be adapted to patients’ clinical needs^40^. Under the conditions of this study, common pure fluoride components initially do not appear to improve the erosion preventive pellicle properties. Cleary, the effects of an iterative and long-term fluoride application as well as a modification of the preparations’ pH values have been excluded in the present study and require future investigation.

## Data Availability

All processed data are provided by this published article. Raw datasets analysed during the current study are available from the corresponding author on reasonable request.
